# Effect of Critical Process Parameters on the Granule Quality During a Binder-Free High-Shear Wet Granulation Process of Mesoporous Silica Microparticles While Achieving Core–Shell Structured Granules

**DOI:** 10.3390/ph19070975

**Published:** 2026-06-23

**Authors:** Flórián Benkő, Nóra Zacsik, Ádám Tóth, Dániel Sebők, Viktória Hornok, László Janovák, Ákos Kukovecz, Tamás Sovány, Katalin Kristó

**Affiliations:** 1 Institute of Pharmaceutical Technology and Regulatory Affairs, University of Szeged, Eötvös u. 6., H-6720 Szeged, Hungary; benko.florian@szte.hu (F.B.); kristo.katalin@szte.hu (K.K.); 2 Department of Applied and Environmental Chemistry, University of Szeged, Rerrich B. tér 1, H-6720 Szeged, Hungarysebokd@chem.u-szeged.hu (D.S.); kakos@chem.u-szeged.hu (Á.K.); 3 MTA-SZTE Momentum Biocolloids Research Group, Department of Physical Chemistry and Materials Science, Interdisciplinary Centre of Excellence, University of Szeged, H-6720 Szeged, Hungary; vhornok@chem.u-szeged.hu; 4 Department of Physical Chemistry and Materials Science, University of Szeged, Rerrich B. tér 1., H-6720 Szeged, Hungary

**Keywords:** mesoporous silica microparticle (MSM), binder-free wet granulation, micro-CT measurements, core–shell structured granules, granule characterization, compressibility study, tablet compression

## Abstract

**Background/Objectives:** The aim of current study was the significant improvement of both the flowability and the compressibility of mesoporous silica microparticles (MSMs), to enable the formulation a potential drug delivery system. MSMs are of emerging interest in the pharmaceutical industry, due to their numerous advantages and versatile applicability, such as improvement in aqueous solubility and epithelial permeability, thus enhancing the oral bioavailability of drugs. However, the formulation of these types of materials has been a major challenge. This problem originates from poor powder flow characteristics due to particle properties. **Methods:** A binder-free high-shear wet granulation (HSWG) process was performed to improve the flowability and compressibility of the model material, meanwhile preserving its porosity. The prepared granules were characterized by particle size, size distribution, yield percentage, particle morphology, porosity, powder flowability, crushing strength, and stability. Micro-CT measurements were performed to examine the structure of the granules and to see the internal segmentation resulted by the two-step granulation process. The granules were compressed into tablets to evaluate the compressibility behavior based on the models of Kawakita and Walker. The physical parameters of the compressed tablets, such as breaking hardness, tensile strength, and thickness, were tested. **Results:** The prepared granules were evaluated successfully according to the mentioned properties and found to be satisfactory compared to the raw materials. The binder-free method appeared to be effective, thus the use of binders may be avoided if the process is designed well and critical process parameters (CPPs) selected carefully. The granules showed good stability over a one-year testing period. The micro-CT test also verified the success of the initial concept of preparing core–shell structured granules, and enabled the determination of macropores. Nevertheless, the results were completed with BET measurements to determine specific surface area of the granules. **Conclusions:** The effect of the critical process parameters of the granulation process on all the mentioned attributes was investigated and since major differences were observed between the batches, the effect of the selected CPPs were also verified.

## 1. Introduction

Mesoporous silica microparticles (MSMs) have emerged as promising candidates as drug delivery systems in the pharmaceutical field [1]. The adjective mesoporous refers to the pore size range of 2–50 nm [2,3,4]. MSMs are efficient drug carriers due to their large absorption capacity, which is advantageous in numerous aspects. The large pore volume and the resulting high specific surface is suitable to bind a big amount of active pharmaceutical ingredients (APIs), while narrow pores can stabilize drug molecules in amorphous form and prevent recrystallization [2,3,4,5]. This effect is called the steric hindrance, where the confined space within the pores prevents the formation of critical mass initiating crystallization [6,7,8]. Keeping the API in amorphous form is highly beneficial due to improved aqueous solubility, which is the main advantage if APIs with poor aqueous solubility are processed [9,10,11,12,13]. The encapsulated drug is also protected against numerous factors, such as chemical or enzymatic degradation [12,14,15], and additionally, an effective masking of bitter taste and unpleasant odor of the API can be achieved [9,16]. These benefits can result in enhanced oral bioavailability [9,10,12,15]. However, this type of material has poor powder flowability and compressibility, due to several causes, such as irregular particle shape, small particle size, low bulk density, high hygroscopicity, and brittleness [3,17,18]. Tahan et al. observed high tendency of particle fragmentation and brittleness, due to the high degree of porosity, which resulted in reduced tensile strength and increased friability in correlation with the microparticle ratio. Nevertheless, the mechanical strength of the tablets was significantly improved by adding additional excipients, such as microcrystalline cellulose to facilitate plastic deformation over fragmentation [18].

The poor flowability is also a major challenge in terms of producing uniform batches and dosing units [19,20]. The granulation of raw materials can be a promising solution to achieve good flow behavior and to overcome uneven die filling and powder segregation at higher tableting speeds, and other difficulties of the processing of mesoporous silica microparticles [18,21,22]. By agglomeration, the desired degree of dispersity may be achieved, so the flowability and compactibility/compressibility (tabletability) can also be improved [17,22]. Nevertheless, granulation may considerably reduce the specific surface area and pore volume, resulting in a decreased drug absorption capacity [17,23], especially if dry granulation is applied, as the well-tuned pore structure may be damaged by the extremely high compression forces and so the drug absorption can be decreased and the expected drug release rate may be modified [10,24,25]. Therefore, wet granulation is the preferred technique to provide the desired flowability while keeping the pore structure intact. Nevertheless, wet granulation can also be associated with reduced absorption capacity if a polymeric binder is used to promote aggregation due to clogging the narrow pores and closing the channels, resulting in a drastic decrease in pore volume and specific surface area [23,26]. Although mesoporous silica was included in different granulation techniques, such as melt-, steam-, or high-shear granulation, as well as extrusion–spheronization, all methods included a binder [17,23,24,27,28].

High-shear wet granulation (HSWG) is a well-known, timesaving, process with exceptional particle quality, and with careful control of CPPs, direct pelletization with uniform distribution can even be achieved [29,30]. HSWG is also used for processing poorly flowable compounds like MSM-based systems and related poorly flowable materials as it can handle a wide variety of raw materials, especially within the Manufacturing Classification System (MCS) framework [31]. HSWG enables the fine-tuning of granule properties by carefully controlling the three phases of granulation e.g., wetting and nucleation, consolidation and growth, attrition and breakage [26]. The most important CPPs are the impeller speed, the chopper speed, the liquid addition rate, and the total amount of granulation liquid, which determine and influence the critical quality attributes (CQAs), such as particle size, size distribution, particle morphology, yield percentage, crushing strength, porosity, friability, flow properties, etc. Some authors found that impeller speed influences friability, particle size, and morphology [32,33,34], and the impeller speed affects all three granulation stages mentioned above [35]. Other authors investigated the effect of chopper speed on particle size and size distribution [33,34,36] by mainly affecting the breakage of granules [34]. The total amount of liquid added influences particle size and size distribution [37], along with the liquid addition rate [38,39], which affects the powder wetting and the granule growth rate [35]. Wet massing time also influences granule strength [40] by affecting the growth and consolidation of the granules [35]. Among these, impeller speed and liquid addition rate were selected for further investigation in the present study, while other parameters were kept constant. The primary aim was to investigate the effect of the previously selected critical process parameters on the critical quality attributes of mesoporous silica microparticles during a binder-free high-shear wet granulation process. The secondary objective was to prepare core–shell granules by a unique granulation approach, in a way that the model MSM forms the core, covered and protected by the MCC shell, allowing binder-free granulation. The tertiary objective of this study was to carry out a compressibility study to justify improved compressibility of the mesoporous carrier by granulating it with microcrystalline cellulose to investigate the effect of the selected critical process parameters on the compressibility and the physical tablet properties.

## 2. Results and Discussion

### 2.1. Particle Size Distribution and Yield Percentage

**Figure 1:**
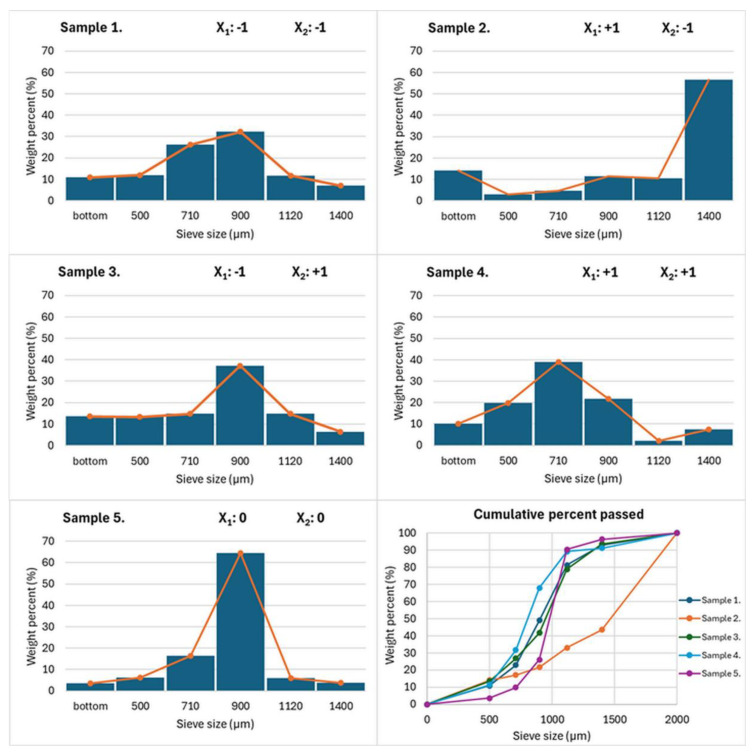
Column charts resulting from sieve analyses, and the cumulative percent passed curves.

The results of sieve analysis (Figure 1) showed high variation of size and size distribution of the granules (Table 1) and the yield percentage (Table 2) of various batches.

The mean particle size was observed to be between 806 and 1468 µm (981 µm, if Sample 2 is excluded as outlier). This particle size range is bigger than what was achieved by Baumgartner et al., who prepared granules with a mean diameter in a range of 207–481 µm. Nevertheless, the difference may be due to lower impeller speeds and water addition rate that did not exceed (50–200 rpm and 4.2 g/min, respectively) in addition to the use of isomalt [23]. A similar tendency could be observed in the study of Habib et al., who wet granulated silicified microcrystalline cellulose and observed a mean particle size of 126 µm and 347 µm at 80% L/S or 100% L/S ratio, respectively, and also applied lower impeller speed (370 rpm) and shorter wet massing time (10 min) [41].

Analysis of the size distribution also showed a considerable variation among different batches, ranging from 0.41–1.05 for Sample 5 and Sample 2, respectively. Nevertheless, these results were more consistent as for Baumgartner et al., who produced granules with higher span values at a range of 1.68–3.05 [23]. The results of two-level full factorial design gave the following equation (Equation (1)) with best-fit for span value (Y_1_). The bold numbers represent the significant factors. Full equations may be found in Supplementary Material.

(1) $$\begin{array}{c} Y_{1}=1.0030+0.0175X_{1}-0.0270{X_{1}X}_{2}\; \end{array}$$

R^2^ = 1; Adjusted R^2^ = 0.99999; MS Residual = 0.000001; Curvature = **−0.5890**

The yield is crucial to develop a cost-effective, robust method. Similarly to the particle size distribution, Sample 2 showed the worst and Sample 5 the best results, where an almost 6-fold improvement could be detected. The results (12–73%) were worse than for Baumgartner et al., who achieved 72–95% yield percentage, but the difference may be explained by the binder-free approach and the more narrow target particle size range in our case [23].

The statistical analysis revealed that the studied factors exerted insignificant (*p* > 0.05) influence on the yield, but impeller speed had a more considerable impact than liquid addition rate as only a sufficiently high mixing speed can prevent unwanted over-wetting of the powder at high liquid addition rates, as was the case in the sample with the second highest yield (Sample 4). The best-fit equation (*p* < 0.05) for yield percentage (Y_2_) was determined based on the highest adjusted R^2^, and lowest MS residual at the same time (Equation (2)).

(2) $$\begin{array}{c} Y_{2}=40.7825+12.7875X_{2}+10.3675{X_{1}X}_{2}\; \end{array}$$

R^2^ = 0.94624; Adjusted R^2^ = 0.78496; MS Residual = 111.408; Curvature = 33.1075

When high liquid addition rate was combined with slow impeller speed (Sample 2), the lowest yield was achieved, as uneven wetting was inevitable due to the lack of proper moisture distribution. The ratio of over- and under-wetted areas was chaotic, irregular, and highly unpredictable. In under-wetted areas the moisture is not sufficient for proper particle agglomeration, providing barely formed granules, or a powder-like mass, while, too much moisture results in larger fragments or the wet mass sticking to the vessel wall, as was observed. However, the highest yield percentage was obtained when both parameters were set to moderate values, as low impeller speed (Samples 2 and 1) provided insufficient mixing ability, but higher impeller (Samples 3 and 4) speed was found to be too drastic, as could be seen in the sequence of the batches [26,35,42].

### 2.2. Particle Morphology

The most important shape descriptors are displayed in Table 3. In case of circularity and roundness, the higher values, while in the case of aspect ratio, the smaller values, represent more spherical particles. In other words, the closer the number is to 1.000, the more spherical the particles are. Microscopic pictures of the various batches may be found in Supplementary Material.

Sample 2 was found to be the least spherical, most elongated, and most irregular among the batches, according to all the shape descriptors. This can be explained by the insufficient and uneven liquid distribution supplemented by the low rate of shear-induced densifying effect and high addition rate (10 mL/min), which lead to insufficient and uneven liquid distribution that negatively affects nuclei formation. Moreover, the lower wet massing time also contributes to insufficient achievement of the equilibrium stage at which a constant amount of liquid is on the surface of particles (the coalescence force is equal to the breaking force) [26]. Furthermore, due to the inadequate agglomeration process, this batch was the most brittle and friable according to the hardness test results (see Section 3.4.4). The high SD values indicate heterogeneous populations, in comparison with other batches, especially Sample 5, which showed the most homogeneous population (Table 1), lowest aspect ratio, and highest roundness, suggesting a more uniform and robust pellet morphology, while Sample 1 exhibited the highest mean circularity, indicating the highest degree of sphericity. Overall, Samples 1 and 5 exhibited a pellet-like morphology, characterized by acceptable circularity (≈0.80), aspect ratio ≤1.2, and high roundness [43,44,45]. Samples 3 and 4 showed intermediate characteristics and were classified as borderline pellets, while Sample 2 showed inferior sphericity and greater shape variability, indicating granule-like morphology [43,44,45].

Thapa et al. prepared granules (MCC, lactose monohydrate) with additional binders, and they observed correlation between the impeller speed and morphology [46]. According to the SEM images granules produced by higher impeller speed were smoother and spherical, while slower impeller speed resulted in rough and irregular granules [46]. But, interestingly, in our case the liquid addition rate played a more important role, especially at slower impeller speed.

From the aspect of CPPs, it can be stated that the liquid addition rate and wet massing time have the most considerable effect over the impeller speed on the particles’ sphericity. Therefore, if an optimal balance is achieved, a higher degree of sphericity is observed in a more homogeneous population.

In HSWG, the link between the liquid addition rate and impeller speed is crucial for process management because both variables work together to influence the severity of collisions, uniformity of wetting, and the subsequent mechanical consolidation of granules. For example, granule consolidation and rounding are encouraged by higher impeller speeds, which typically increase the mechanical energy input into the bed. Higher shear forces efficiently ‘polish’ the granules against the vessel wall at high speeds, increasing sphericity if the granules are not excessively friable. In addition, the rate of liquid addition determines the uniformity of the wet mass. If the liquid addition rate is too high relative to the impeller speed, it can lead to localized over-wetting, where excessive binder concentration on specific particle surfaces causes rapid, nonuniform growth, and results irregular, non-spherical shapes. Optimal sphericity is achieved when the shear intensity (impeller speed) is high enough to consolidate the particles, while the wetting rate is controlled to ensure that the material remains plastic enough to deform into spherical shape without triggering excessive coalescence into irregular agglomerates [47].

### 2.3. Powder Flowability

Good flowability is crucial to enable uniformity of mass and drug distribution in tablets. The data are summarized in Table 4.

When comparing the indicators of powder flowability, an enormous improvement was observed in case of the granules, in comparison with the raw materials. Both raw materials showed poor flowability, especially MSM, the key material. When comparing granulated batches, slight differences could be observed, with Sample 5 as the best and Sample 2 as the worst, findings that are consistent with the results of the morphological analysis.

The obtained results are better than those achieved by Baumgartner et al. (Hausner ratio between 1.18–1.41) or Thapa et al. (Carr’s index between 9.28–16.90), which may be due to the bigger particle size and favorable particle shape of our products [23,46].

During the granulation process, the key was to balance the liquid addition rate with the mixing capacity of the impeller (impeller speed), resulting in uniform liquid distribution and, thus, optimal particle characteristics.

### 2.4. Micro-Computed Tomography (Micro-CT) Measurements

**Figure 2:**
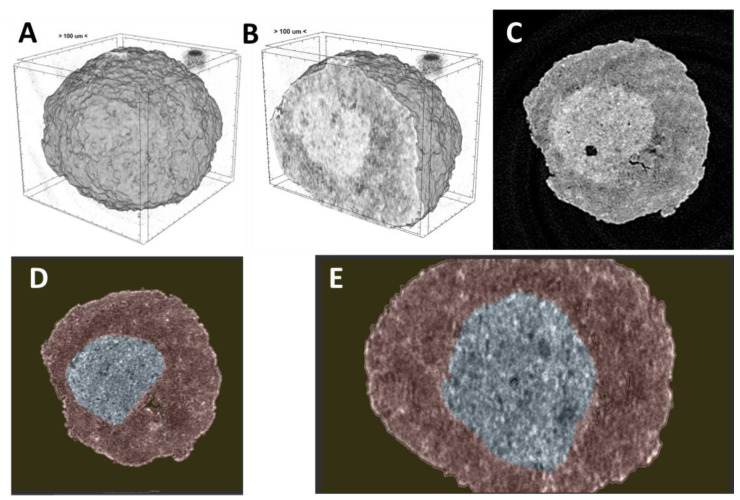
Micro-CT images of Sample 5. (**A**): 3D image on the intact particle; (**B**): 3D cross-cut image (**C**): 2D cross-cut image, representing an individual slice; (**D**,**E**): AI segmentation on a 2D cross-cut image from two different projections (blue: internal core, red: outer shell).

The micro-CT measurement revealed the details of the internal structure of the granules, which would remain invisible using only traditional characterization methods. Due to the two-step granulation process, the granules showed a core–shell structure (Figure 2). The silica formed the inner core of the particles, while MCC formed an outer shell on these cores during the second-phase of granulation. In this case, the brittle silica, which hardly forms regular granules due to weak bond formation properties, was locked inside and surrounded by the protective layer of the denser and better bond-forming MCC shell. Figure 2D shows the internal structure of the particle after visualization with an AI-based segmentation technology, verifying the successful preparation of the core–shell model. The inner blue color represents the silica core, and the outer red color represents the MCC shell. The sharp boundary is clearly visible between the two layers of materials. The hardly penetrable, compact MCC shell might isolate the MSM core from external impacts and, thus, might increase the stability of MSM [48].

The micro-CT analysis is also suitable to determine general porosity of the granules by analyzing each slice and calculating the total porosity by combining the slices. Nevertheless, it should be noted, that the resolution of this technique is limited at this particle size (3 µm/pixel), which only enabled the determination of macropores over this size. The corresponding porosity values can be seen in the following table (Table 5).

The results are relatively small compared with literature data, as Kašpar et al. obtained porosity values between 7–18% measured at 3.8 μm resolution [49]. Nevertheless, in their study, the starting particle size of raw materials was bigger and lower impeller speed was applied, which may induce lower shear stress and reduced deformation, and, thus, explain the formation of bigger pores which are better visible for the micro-CT. Similarly, Rahmanian et al. obtained high porosity values at 0.87–2 μm resolution [50], and compared the results with mercury porosimetry and found that the porosity values calculated based on micro-CT results were higher, since closed pores were also visible. However, they used a less ductile starting material and smaller shear stress, which resulted in less dense traditional granules. In our case, the obtained low porosity results, can be explained by the high shear stress induced by the high impeller speeds, which caused intensive densification of MCC particles and resulted in a pellet-like texture, while extremely small particle size of the mesoporous silica did not enable the visual identification of the pores at the obtainable 3 μm resolution of micro-CT images. Thus, our results are in better accordance with the results of Matsui et al., who examined particles produced by extrusion and found that micro-CT provided smaller porosity values than mercury porosimetry due to the compact texture of the particles and the 6 μm resolution, so smaller pores were not visible on micro-CT images [51].

The two-level full factorial design results were analyzed, and the best-fit equation (*p* < 0.05) for porosity (Y_3_) was determined based on the highest adjusted R^2^, and lowest MS residual at the same time (Equation (3)). The bold numbers represent the significant factors.

(3) $$\begin{array}{c} Y_{3}=0.6650-0.3925X_{2}+0.0495{X_{1}X}_{2} \end{array}$$

R^2^ = 0.99975; Adjusted R^2^ = 0.99901; MS Residual = 0.000196; Curvature = **−0.4570**

**Figure 3:**
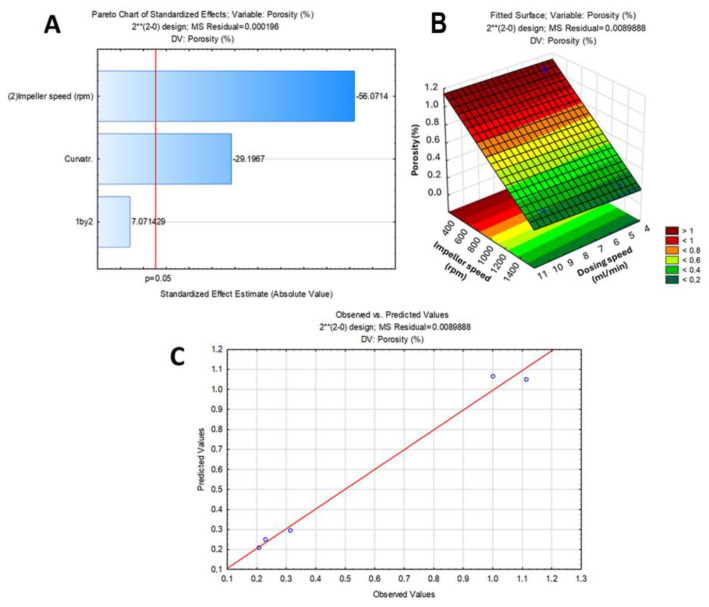
Statistical analysis results of the porosity tests. (**A**): Pareto chart; (**B**): Surface plot; (**C**): Observed vs. Predicted curve.

It is clearly visible that despite the good fit, a significant nonlinearity may be detected in the results. The statistical analysis showed that the impeller speed has a significant effect on the porosity of the granules (Figure 3), which can be associated with a more intensive densifying effect of the impeller at higher speeds. Although the liquid addition rate seemed to have only a negligible effect on the porosity. Higher porosity values were observed in case of the batches prepared at slower or moderate impeller speeds; in contrast, a decreased porosity was achieved at higher speeds.

To overcome the limitations of µCT measurements, the results were completed with BET measurements, which enabled the determination of the specific surface area of the granules (Table 6).

The results justified, that despite of the low apparent porosity of the granules based on image analysis, considering the composition of granules the MSM have mostly preserved their original porosity, and the obtained samples are suitable for drug binding purposes.

### 2.5. Crushing Strength and Mechanical Properties

The crushing strength of the prepared granules was determined based on the measurement of 40 granules of each batch (Table 7). The breaking force values of the granules varied between 3.8 and 11.2 N, and there is no existing publication about granulating silica, testing the strength of the granules.

**Figure 4:**
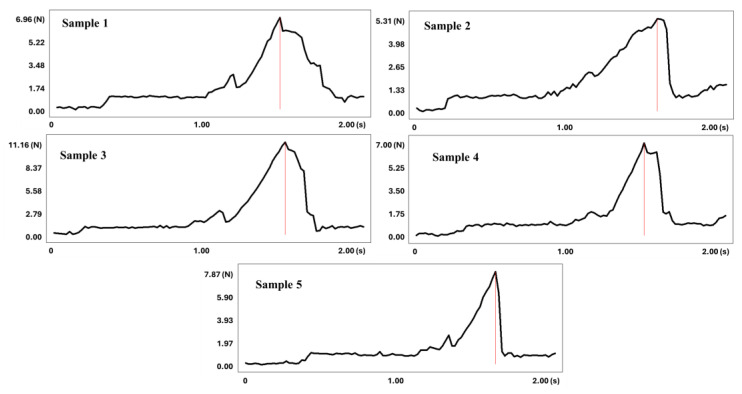
Deformation curves. Each batch is represented by an example curve.

The representative deformation curves of each batch are displayed in Figure 4. It is well visible that the granules undergo on a multistep breaking process, starting with a long viscoelastic phase possibly due to the deformation of the outer MCC layer, followed by a small drop of the breaking force which possibly may indicate the formation of microfractures in the interfacial layer. The final step is an intensive deformation followed by the cracking of the granule.

After statistical analysis (Equation (4)), none of the selected factors exerted significant effect on granule hardness (Y_4_), but the impeller speed had more considerable impact compared to liquid addition rate.

(4) $$\begin{array}{c} Y_{4}=6.6016-1.4345X_{1}+2.3105X_{2}-1.043X_{1}X_{2}\; \end{array}$$

R^2^ = 0.99395; Adjusted R^2^ = 0.97581; MS Residual = 0.2064512

**Figure 5:**
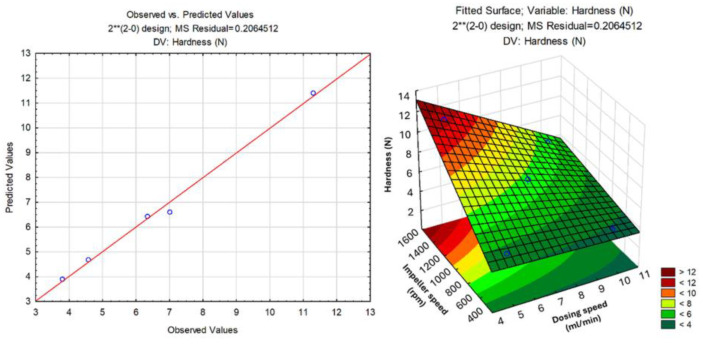
The results of the statistical analysis of the breaking hardness tests. (**Left**): Observed vs. predicted curve, (**Right**): Surface plot.

This phenomenon can be explained by the densification effect of the impeller, since at higher speeds the impeller not only mixes the wet mass but compacts (squeezes) it as well, which resulted in the formation of more compact agglomerates. This is consistent with the results of previous studies, where impeller speed was in direct proportion with granule hardness, as it plays a vital role in creating cohesive force ensuring uniform liquid distribution and enhancing particle agglomeration [52], due to higher torque, basically densifying the wet mass [52]. This is also supported by the porosity results from µCT analysis. Nevertheless, upon comparison of the results at the same impeller speed, granules with better mechanical properties were prepared at a slower liquid addition rate, as fast liquid addition may lead to ununiform distribution, resulting in over- or under-wetted parts. Combined with low impeller speed, this means improper agglomeration in terms of granule integrity. The visualizations from the statistical analysis can be seen in Figure 5.

The observed–predicted curve shows great reproducibility, and the decent fitting means a robust experiment.

### 2.6. Moisture Content Analysis

The mesoporous material has a large specific surface area, with plenty of possible binding sites. Our research focused on a binder-free approach of granulation processes to avoid the clogging of narrow pores. The moisture content of the granules is an indirect indicator of the intactness of the carrier internal surface and of the permeability of the pores for possible API absorption. Water molecules and drug molecules compete for the same binding sites inside the pores (primarily surface silanol/OH groups), meaning lower moisture content means more open pores, more free binding sites, and, thus, a larger surface area for drug absorption [53,54,55]. This indirect relation is especially important since that type of material is highly hygroscopic and readily absorbs moisture from the air. Therefore, the goal was to keep the moisture content to a minimum. The following Table shows the moisture content determined after preparation (after drying) and after a 1-year period, in all five cases (Table 8).

It is clearly visible that the moisture content of all granules was lower than the moisture content of raw materials alone, MSM (≈11.81%) and MCC (≈4.56%), or the average moisture content of the 1:1 powder mixture after the same pretreatment (drying). This supports the hypothesis that despite the large amount of added moisture, the subsequent drying step was effective, and since no polymeric binder was added to aggregate the particles, the clogging of narrow pores was prevented. Furthermore, as only a slight increase was observed during the 1-year stability testing period, it can be stated that the outer MCC shell can prevent the excess moisture uptake of the MSM core.

### 2.7. Compressibility Study

**Figure 6:**
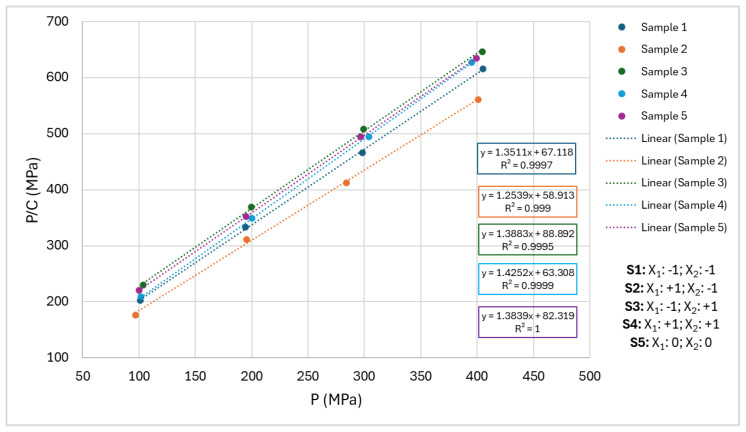
Kawakita plot calculated with the in-the-die method based on Equation (11).

**Figure 7:**
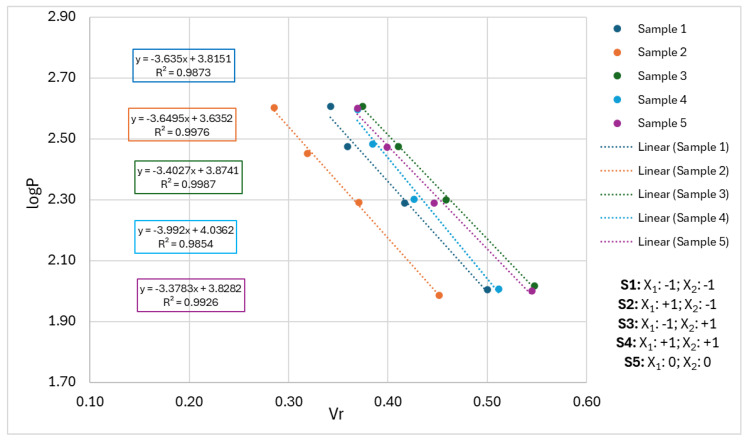
Walker plot based on Equation (13).

**Figure 8:**
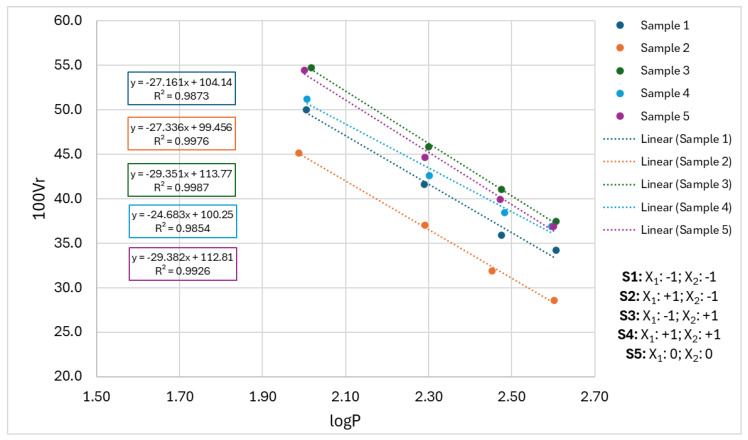
Walker plot based on Equation (14).

The secondary aim of this work was to study the compactibility (tensile strength as a function of the tablet porosity) and compressibility (tablet porosity/ volume reduction as a function of the applied compression stress) behavior of the granules. The following Figures show the plots created based on the method of Kawakita and Lüdde (Figure 6) and Walker (Figure 7 and Figure 8). After the visual representation of the compressibility results, the calculated coefficients and constants are displayed in Table 9.

Using the Kawakita–Lüdde model, the densification behavior and maximum compactibility of different powders during the rearrangement phase of the tablet compression can be quantified and compared. The coefficient *a* refers to the minimum porosity of the powder, higher values mean better compactibility and higher initial porosity. Coefficient *1/b* refers to the stress needed to reduce the initial volume of powder bed to its half, it is determined by the degree of plasticity and cohesiveness, or can refer to the fragmentation behavior of the granules, thus lower *1/b* means easier densification, higher plasticity, and the material compacts and deforms easily [56,57,58,59].

The slight differences between batches are mostly consistent with the hardness test results and the process variables. Harder samples, which were prepared at high impeller speed and have decent compactness, reached the lowest theoretical value of maximal volume reduction. In contrast, more friable and less compact samples are associated with a high degree of maximal volume reduction, and require the least energy to rearrange and densify.

In summary, Kawakita constants followed the expected order, proving a strong positive correlation between the crushing strength and compatibility of the granules.

The results are consistent with the observation of Thapa et al., who investigated the compressibility of granules by the model of Kawakita [46]. The constant *a* and *1/b* in their case varied between 0.64–0.69 and 52–160 MPa, respectively. The better compressibility and higher degree of volume reduction in our case can be attributed to bigger void spaces due to the bigger particle size of our samples [46].

The compressibility of particles during the main compaction/deformation phase can be compared and quantified using the models of Walker. The coefficients suggested a decent compressibility in the case of all batches. Nevertheless, higher values of coefficient *L*, which refers to the volume of powder bed at a given pressure, are associated with friable samples. Oppositely, coefficient *W* refers to the percentage volume reduction if the compression stress is increased logarithmically is higher for more compact samples, indicating the presence of good bonding behavior and the domination of plastic deformation over elastic ones, or fragmentation [56,60,61].

### 2.8. Physical Properties of Tablets

**Figure 9:**
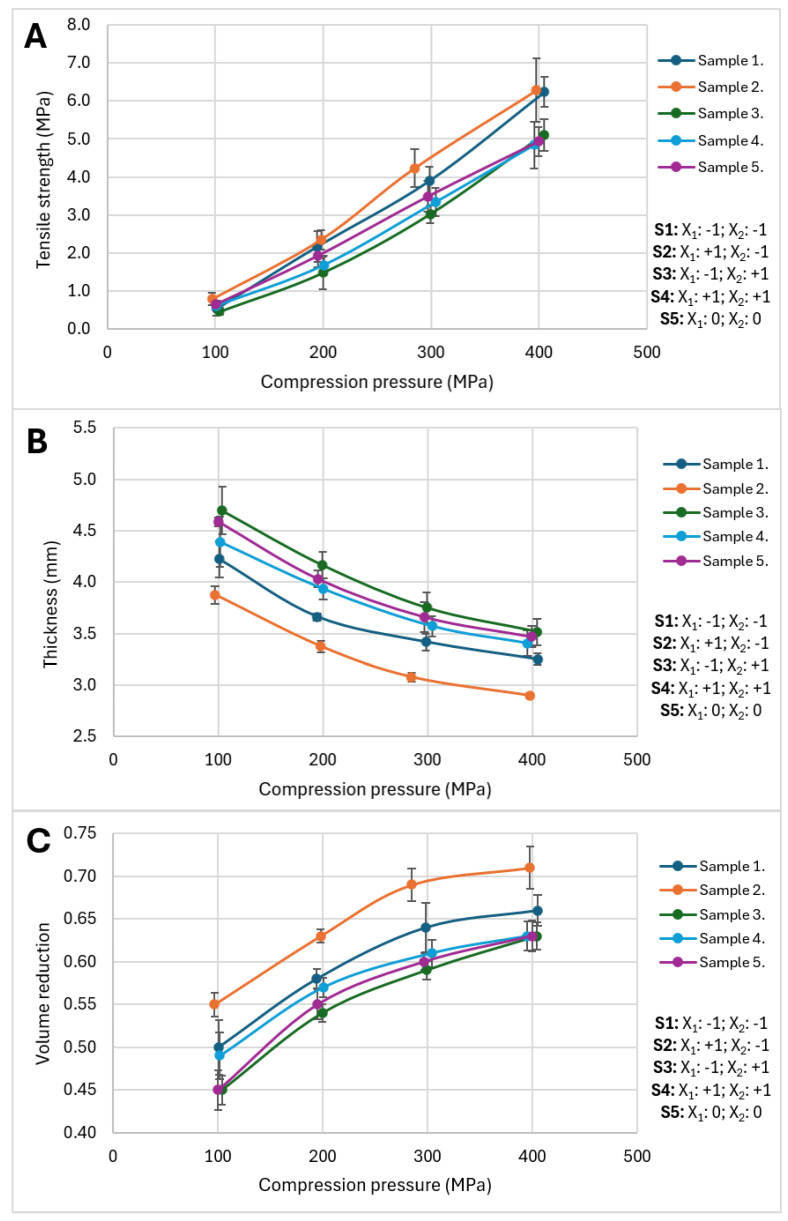
Physical tablet properties (**A**): Correlation between the tensile strength and the compression pressure (Tabletability). (**B**): Relationship between the thickness of the tablets and the compression pressure. (**C**): Correlation between the apparent in-die volume reduction and the compression pressure.

After the tablets were compressed, another test sequence was conducted to characterize their physical properties (breaking hardness, tensile strength, thickness) in correlation with the applied pressure. The data are displayed on Figure 9.

Figure 9A shows the compression stress dependence of tensile strength. An increase in the tensile strength of compacted materials corresponds to an increase in bond strength per unit area [56]. Although at a compression stress of 100 MPa, only small differences were observed between the different batches, the difference increased continuously with the increasing compression pressure. At high pressures, samples were separated into groups based on process variables, mainly on the impeller speed. Samples prepared at lower impeller speed thus had higher porosity and friability and provided more robust tablets. In contrast, more compact and harder particles obtained at high impeller speeds require more energy to be deformed to create new interparticle bonds. However, despite the differences, all batches provided decent hardness and tensile strength, even at lower pressures, which suggests that plastic deformation and irreversible bond formation occur also at lower pressures, facilitating robust tablet compression and excellent tablet integrity.

Figure 9B shows the correlation between compression force and thickness. At the beginning, a relatively intensive decrease can be observed, with the increasing pressure, but at higher pressure ranges, the decrease of thickness is more moderate as the powders reach the theoretical limit in volume reduction, as determined by the Kawakita model. However, the Kawakita model was calculated by the in-the-die method, relying purely on the densification behavior of the granules, but by measuring the thickness after compression, the elastic recovery of the material also should be considered, which explains the differences between the behavior of batches, comparing the two approaches. Nevertheless, the main trends and the order of the batches are in good correlation. In addition to that, for the highest granule crushing strengths, the highest tablet thickness was obtained, and vice versa—which is also in accordance with results from literature [62,63]. In this case, the order of the batches is completely identical from both perspectives, and the results justify the previous observation that a more compact, dense granule is only capable of a limited volume reduction, compared to the less compact, less dense particles, where the decrease in thickness is more pronounced.

Figure 9C displays the apparent in-die volume reduction of the prepared granules. An increased volume reduction can be observed with increasing compression pressure, but the extent of reduction keeps decreasing at higher pressures as the deformation of granules exceeds the elastic limit. Sample 2 shows the highest volume reduction, which can be explained by the hardness and porosity values. This sample appeared to be the least compact and the most brittle, resulting in the highest volume reduction during tablet compression. In contrast, sample 3, which appeared to be the most dense and compact and had the highest hardness values, and thus is hardly deformable, which leaves less room for volume reduction after particle rearrangement.

## 3. Materials and Methods

### 3.1. Chemicals

In this experiment, mesoporous magnesium aluminometasilicate (Neusilin^®^ FH1, Fuji Chemical Industries Co., Ltd., Osaka, Japan) (specific surface area ≈ 110 m^2^/g, mean particle size ≈ 70–110 µm (primary agglomerates)) was used as mesoporous silica microparticle (MSM), microcrystalline cellulose (MCC) (Comprecel^®^ 101, Ming Thai Chemical Co., Ltd., Taoyuan City, Taiwan) (specific surface area ≈ 2.5 m^2^/g, mean particle size ≈ 50 µm) was used to improve the hardness and compressibility of granules. The materials were used as received and purified water was used as the granulation liquid.

### 3.2. Preparation of the Granules

The granulation process was carried out with a ProCepT 4M8 high shear granulator (ProCepT nv., Zele, Belgium). The investigated factors (impeller speed and the liquid addition rate) were set to the selected values based on the created experimental design. At the same time, the remaining factors (chopper speed and total amount of water added) were kept constant at an optimal value based on the result of preliminary experiments. The preparation method, which was designed based on preliminary experiments, was the following.

First, 50 g of the MSM was added to the granulation chamber (based on the filling capacity of the glass chamber), and 40 g of liquid was added to the powder in two portions (20 + 20 g), keeping a 2 min kneading time after the dosing of each portion. After the second kneading, 50 g of MCC was added to the wet mass to obtain 1:1 mass ratio of dry materials. Next, another 20 g of water was added to the system, followed by another 2 min of kneading. Finally, extra 30 g of liquid was added, and production ended with the final 2 min kneading step. The kneading times mentioned were selected to give enough time to absorb the moisture, while distributing evenly. The liquid/solid mass ratio (L/S) was also kept constant at a value of 9/10. The prepared granules were collected and dried in a drying oven (Memmert GmbH + Co. KG, Schwabach, Germany) at 50 °C for 3 h.

### 3.3. Design of Experiments (DoE)

Statistical analysis was performed using the Statistica^®^ program, version 13 (TIBCO Software Inc., Palo Alto, CA, USA). The CPPs, impeller speed (x_1_) and the liquid addition rate (x_2_), and their corresponding factor levels are displayed in Table 10. The levels were selected based on preliminary experiments and literature review. The optimization was based on a two-level full factorial design with a central point and a total of 5 runs (Table 11).

A statistical model was fitted to the results, the general equation of which is as follows:

Y = b_0_+ b_1_X_1_ + b_2_X_2_ + b_12_X_1_X_2_

where b_0_ is the intercept, i.e., the overall mean of the results, b_1_ is the coefficient for the liquid addition rate, b_2_ for the impeller speed, and b_12_ for the interaction between the two factors. Coefficients are referring to the change in the optimization parameter if the value of the corresponding factor is raised from the 0 to the +1 level.

### 3.4. Characterization of the Granules

#### 3.4.1. Particle Distribution and Yield Percentage

The prepared granules were sieved with a vibrational sieve (Retsch GmbH, Haan, Germany) for 20 min and subsequently analyzed to determine the mean particle size, the size distribution, and the yield percentage at the same time. The sieve set contained sieves with the following mesh sizes: 500 µm, 710 µm, 900 µm, 1120 µm, 1400 µm, 2000 µm. The sample amount in each sieve was weighed, and the total sample percentage between 710 µm and 1120 µm was recorded. The preferred particle size range was 710–1120 µm, considering the optimal die filling while maintaining the good flow characteristics. Any sieve fraction below this range cannot be considered as well granulated; therefore, enhanced flowability was not shown, and uniform die filling was not provided. In contrast, the size fraction above the chosen range seemed to be oversized for optimal die filling. Everything greater than 2000 µm in size was disposed as process waste. The yield percentage was calculated according to the following equation (Equation (5)), where the final granules weight was considered for granules in the preferred size interval. The characteristic particle sizes d (0.1), d (0.5), and d (0.9) were determined from the sieve analysis results using the cumulative particle size distribution curve. These values correspond to the particle diameters at which 10%, 50%, and 90% of the sample mass passed through the sieves, respectively, and were obtained by interpolation between the measured data points. For describing the size distribution, the span value was calculated based on the following equation (Equation (6)).

(5) $$\begin{array}{c} Yield\;percentage\;\left(\%\right)=\frac{Final\;granules\;weight\;\left(g\right)}{Initial\;powder\;weight\;\left(g\right)}\times 100\; \end{array}$$

(6) $$\begin{array}{c} Span=\frac{d\left(0.9\right)-d\left(0.1\right)}{d\left(0.5\right)}\; \end{array}$$

#### 3.4.2. Particle Morphology

Aspect ratio (AR), roundness, and circularity of 300 granules were determined using a stereomicroscope (Carl Zeiss, Oberkochen, Germany). The main shape descriptors were calculated with an image processing software (Fiji-ImageJ, version 1.53c, National Institute of Health, Bethesda, MD, USA), according to the following equations (Equations (7)–(9)). After calculating, the mean and standard deviation values of the various batches were compared.

(7) $$\begin{array}{c} Aspect\;ratio\;(AR)=\frac{{Diameter}_{max}(Major\;axis)}{{Diameter}_{min}(Minor\;axis)}\; \end{array}$$

(8) $$\begin{array}{c} Roundness=\frac{4\times Area}{\pi \times {Major\;axis}^{2}} \end{array}$$

(9) $$\begin{array}{c} Circularity=\frac{4\pi \times Area}{{Perimeter}^{2}} \end{array}$$

#### 3.4.3. Powder Flowability

The powder flowability was tested using two different devices. Bulk density, tapped density, Hausner ratio, and Carr index were determined by using a TD-1 tap density tester (Sotax AG, Basel, Switzerland). The volume values were recorded after defined tap numbers, e.g., after 10, 500, 1250 taps. The tapping speed was set to 250 taps/min. The angle of repose was tested by using a PF1 powder flow tester (Sotax AG, Basel, Switzerland). The main flowability descriptors were calculated based on 3 parallel measurements.

#### 3.4.4. Crushing Strength and Mechanical Properties

The breaking force and deformation characteristics of the granules were measured by a laboratory-constructed texture analyzer that contained a measuring probe working at a speed of 20 mm/min, applying a force range of 0–50 N, a sensitivity of ±0.5% ± 0.1 digit, and with an output of 0–5 V. A total of 40 granules were tested in a size range between 710 µm and 1120 µm. The mean and the standard deviation values were calculated.

#### 3.4.5. Micro-Computed Tomography (Micro-CT) Measurements

Structural and morphological characterization of the pellets was performed using micro-CT technique (high-resolution computed tomography) (TESCAN UniTOM XL Spectral, TESCAN, Brno, Czech Republic). The measurements were carried out at 70 kV tube voltage and 15 W tube power with a pixel resolution of 3 μm and an exposure time of 250 ms. The measurement was made of 1737 projection images, which were obtained by a 360° rotation of the sample with 0.207° rotation step in 38 min of scan time. After, image reconstruction and volume-rendered 3D visualization was done with Panthera 2024 (TESCAN, Czech Republic) software and VGSTUDIO MAX v2023.4.0 (Volume Graphics, Germany).

#### 3.4.6. Specific Surface Area Measurements

Specific surface areas were determined by N2 adsorption at 77 K with a Micromeritics Gemini 2375 Surface Area Analyzer device (Micromeritrics, Eindhoven, The Netherlands).

#### 3.4.7. Moisture Content Analysis

The moisture content of the granules was determined using a halogen moisture analyzer (MAC 50/NH, RADWAG, Radom, Poland). Approximately 1 g of granules was examined in each batch. The duration of the test was 20 min at the drying temperature of 120 °C in standard drying mode. The results were expressed in percentage loss of mass (%^M^), and the calculation can be seen in the following equation (Equation (10)). In addition to that, a 1-year stability test was conducted at room temperature (25 ± 3 °C) at 60 ± 5% relative humidity.

(10) $$\begin{array}{c} {\%}^{M}=\frac{Weight\;of\;moisture}{Total\;weight\left(Wet\;weight\right)}\times 100\; \end{array}$$

#### 3.4.8. Compressibility Study

A Korsch EK0 (Korsch AG, Berlin, Germany) eccentric press mounted with strain gauges and with a displacement transducer on the upper and lower punch was applied for tablet compression, with a beveled edge and bisected punches of 8 mm diameter. Each batch was compressed with four different compression forces (5 kN, 10 kN, 15 kN, 20 kN). By collecting the recorded data from the strain gauges and the displacement transducer, a compressibility study was carried out according to the models of Kawakita and Lüdde (Equations (11) and (12)) and Walker (Equations (13) and (14)) [64,65].

(11) $$\begin{array}{c} \frac{P}{C}=\frac{1}{a}P+\frac{1}{ab}\; \end{array}$$

(12) $$\begin{array}{c} C=\frac{V_{0}-V}{V_{0}}\; \end{array}$$

Equation (11). describes the behavior of the powder during the initial phase of the compression, where *a* and *1/b* are constants representing compressibility and cohesiveness, respectively. The letter “a” represents the maximum compressibility, the maximum degree of volume reduction (it is the theoretical limit of volume reduction, the theoretical limit of C, extrapolating to the maximal pressure), reflecting the inherent compactibility of the powder. *1/b* (cohesiveness) is the pressure value required to reach half of the maximum volume reduction in MPa. These constants were determined from the dataset at compression forces mentioned above and plotted according to the in-the-die method. This method means the technique, when compressibility is measured during compression, inside the die. The fractional volume reduction, marked with C, can be calculated using Equation (12), where V is the volume of the powder bed at maximum pressure (marked with P, representing the applied pressure in MPa) and V_0_ is the initial apparent volume of the powder bed.

(13) $$\begin{array}{c} logP=-LV_{r}+C_{1}\; \end{array}$$

(14) $$\begin{array}{c} 100V_{r}=-WlogP+C_{2}\; \end{array}$$

In Equations (13) and (14), P represents the applied pressure, V_r_ represents the relative volume of the material (calculated as V/V_0_, meaning that the volume at the maximum applied pressure divided by the initial volume, resulting in the relative, or normalized volume). C_1_ and C_2_ are constants, as well as the intercept on the plot, when visualizing the results. The letter L is the compressibility coefficient, called the pressing modulus (Walker constant), which means the volume reduction per unit increase on the logP scale. The letter W is the compressibility coefficient, which means the percentage of volume reduction when the applied pressure changes on a logarithmic scale.

#### 3.4.9. Physical Tablet Properties

The tablets were tested using a MT-50 tablet testing instrument (Sotax AG, Basel, Switzerland). Thickness, diameter and breaking hardness were tested for 7 tablets of each compression force. Additionally, tensile strength was calculated using Equation (15) [22,66].

(15) $$\begin{array}{c} Tensile\;strength=\frac{2F}{\pi DH}\; \end{array}$$

where F is the breaking hardness (N), D is the diameter of the tablets (mm), and H is the thickness of the tablets (mm).

## 4. Conclusions

Mesoporous silica microparticles were successfully wet granulated by applying a binder-free approach using a laboratory-scale high-shear granulator with the expectation of better flowability and compressibility of the material. Microcrystalline cellulose was incorporated into the formulation to improve the structural integrity of the granules and to further improve the deformation characteristics of the granules during tablet compression by shifting the deformation characteristics of the mesoporous carrier from particle fragmentation to plastic deformation dominant, enhancing the compressibility behavior. The prepared granules were evaluated for particle and bulk properties. The granules were also tested by using a micro-CT apparatus to investigate the internal structure of the particles and make the core–shell model visible by using an AI-based segmentation method. Moreover, the numerous scans led us to determine the macropores and cavities inside the granules. A compressibility study was performed using the equations of Kawakita and Walker. After that, the compressed tablets were also tested for mechanical properties. The effect of the selected critical parameters (liquid addition rate, impeller speed) was investigated in the case of the mentioned attributes, and these were found to be affected by the parameters to a higher or lesser degree. The granules were successfully prepared with efficient properties, such as aspect ratio < 1.2, crushing strength > 11 N, Span value < 0.5, yield percentage > 73% in the selected size range, acceptable compressibility, and good stability after 1 year. On top of that, excellent flowability was achieved, despite the poor flow properties of the raw materials. Overall, the hardness, flowability, morphology, size distribution, and yield percentage were observed to be acceptable in some batches, while other batches failed one or more properties, proving the importance of the careful selection of the CPPs. In this specific experiment, regarding most of the investigated properties, the best result was observed in the combination of moderate impeller speed (1000 rpm) and moderate liquid addition rate (7.5 mL/min). Moreover, the micro-CT scans provided evidence of the successful application of the current granulation method, which created the core–shell model, as planned.

After optimizing the formulation and the process parameters, in the future we plan to incorporate a model drug and investigate the effect of drug loading on the characterized properties. In addition to that, further characterization methods can be conducted, such as, drug loading efficiency, encapsulation efficiency, drug content, and a dissolution test. Furthermore, the amorphization capacity of the mesoporous drug carrier could be tested, improving the oral bioavailability.

## Figures and Tables

**Table 1:** Particle size distribution indicators.

	Sample 1	Sample 2	Sample 3	Sample 4	Sample 5
d (0.1) (µm)	459.0	355.	369.5	441.2	712.4
d (0.5) (µm)	906.7	1468.9	949.5	806.2	981.7
d (0.9) (µm)	1327.8	1893.8	1331.3	1242.3	1118.5
Span	0.958	1.047	1.013	0.994	0.414

**Table 2:** Yield percentage results in the selected size fraction (710–1120 µm).

	Sample 1	Sample 2	Sample 3	Sample 4	Sample 5
Yield percentage (%)	43.6	12.4	48.5	58.7	73.9

**Table 3:** Results from the morphological analysis for each batch, testing 300 particles.

Shape Descriptors		Sample 1	Sample 2	Sample 3	Sample 4	Sample 5
Circularity	Mean	0.816	0.747	0.778	0.766	0.802
	SD	0.0410	0.0610	0.0396	0.0513	0.0336
Aspect ratio	Mean	1.187	1.308	1.205	1.201	1.161
	SD	0.1173	0.4605	0.1375	0.1308	0.1042
Roundness	Mean	0.850	0.793	0.840	0.842	0.868
	SD	0.0783	0.1099	0.0853	0.0829	0.0711

**Table 4:** Flowability results for the prepared granules, in comparison with the raw materials.

	Sample 1	Sample 2	Sample 3	Sample 4	Sample 5	Neusilin FH1	MCC101
Bulk density (g/mL)	0.551	0.500	0.613	0.577	0.593	0.085	0.323
SD	0.0240	0.0156	0.0099	0.0191	0.0304	0.0042	0.0304
Tapped density (g/mL)	0.624	0.569	0.679	0.647	0.642	0.135	0.445
SD	0.0233	0.0141	0.0042	0.0148	0.0276	0.0057	0.0389
Hausner ratio	1.132	1.138	1.106	1.123	1.082	1.588	1.375
SD	0.0063	0.0068	0.0107	0.0119	0.0097	0.0137	0.0107
Carr’s index	11.63	12.12	9.62	10.94	7.61	37.037	27.416
SD	0.4745	0.5313	0.8860	09344	0.8181	0.5362	0.5582
Angle of repose (°)	30.65	32.54	32.21	31.81	31.22	45	36.27
SD	0.1061	0.3041	0.6293	0.5020	0.1980	0.7071	0.3041

**Table 5:** Porosity values determined by micro-CT measurements and the corresponding standard deviation values.

	Sample 1	Sample 2	Sample 3	Sample 4	Sample 5
Porosity (%)	1.114	1.001	0.230	0.315	0.208
SD (%)	0.079	0.121	0.009	0.112	0.011

**Table 6:** Specific surface area of samples based on BET measurements (n = 3).

	Sample 1	Sample 2	Sample 3	Sample 4	Sample 5	Neusilin FH1	MCC101
Specific surface area (m^2^/g)	28.98	34.88	32.70	34.73	34.66	110 *	2.5 *
SD	0.23	0.34	0.26	0.30	0.31		

* Approximate values obtained from supplier.

**Table 7:** The calculated mean and standard deviation values of each sample, based on 40 granules.

	Sample 1	Sample 2	Sample 3	Sample 4	Sample 5
Mean breaking force (N)	4.58	3.80	11.29	6.33	7.01
SD	1.582	1.426	2.394	2.135	1.436

**Table 8:** Change in moisture content over a 1-year period for each sample.

Moisture Content (%)	Sample 1	Sample 2	Sample 3	Sample 4	Sample 5
After preparation	7.06	8.00	7.58	7.54	7.56
After 1 year	7.23	8.32	7.60	7.92	7.59

**Table 9:** Kawakita and Walker constants and coefficients are calculated from the plots.

	Kawakita		Walker			
	a	1/b (MPa)	L	W	C_1_	C_2_
Sample 1	0.74	49.68	3.88	27.16	3.91	104.1
Sample 2	0.80	46.98	3.59	27.34	3.61	99.46
Sample 3	0.72	64.03	3.30	29.35	3.82	113.8
Sample 4	0.70	44.42	3.56	24.68	3.85	100.3
Sample 5	0.72	59.48	3.47	29.38	3.87	112.8

**Table 10:** Selected CPPs with high, center, and low levels and their coded values.

Factor	Factor Names	Low Level (−1)	Center Level (0)	High Level (+1)
X_1_	Liquid addition rate (mL/min)	5	7.5	10
X_2_	Impeller speed (rpm)	500	1000	1500

**Table 11:** Two factors at two-level full factorial design with a central point for process optimization, supplemented with the constant parameters, including the wet massing times.

Factor	Sample 1	Sample 2	Sample 3	Sample 4	Sample 5
Liquid addition rate (mL/min)	5	10	5	10	7.5
Impeller speed (rpm)	500	500	1500	1500	1000
Chopper speed (rpm)	3000	3000	3000	3000	3000
Total amount of water (g)	90	90	90	90	90
Wet massing time (min)	26	17	26	17	20

## Data Availability

The datasets presented in this article are not readily available because the data are part of an ongoing study that requires further time until the raw data can be transformed to publicly accessible file formats, and the file names and nomenclature used in the various measurements would be unified and the correct metadata could be provided to enable the correct submission of the raw files into a publicly available data repository. Until that time, requests to access the datasets should be directed to the corresponding author (sovany.tamas@szte.hu).

## Supplementary Materials

The following supporting information can be downloaded at: https://www.mdpi.com/article/10.3390/ph19070975/s1, Table S1. Complete equations for the studied independent variables; Figure S1. Microscopical images of Sample 1; Figure S2. Microscopical images of Sample 2; Figure S3. Microscopical images of Sample 3; Figure S4. Microscopical images of Sample 4; Figure S5. Microscopical images of Sample 5; Figure S6. Micro-CT images of granules from Sample 1; Figure S7. Micro-CT images of granules from Sample 2; Figure S8. Micro-CT images of granules from Sample 3; Figure S9. Micro-CT images of granules from Sample 4; Figure S10. Micro-CT images of granules from Sample 5.
