# Supplementary material for: Effect of Critical Process Parameters on the Granule Quality During a Binder-Free High-Shear Wet Granulation Process of Mesoporous Silica Microparticles While Achieving Core–Shell Structured Granules
Source: Pharmaceuticals (Basel). 2026 Jun 23;19(7):975. doi: 10.3390/ph19070975 (PMC13414584; doi:10.3390/ph19070975)
Supplement: Supplementary file 1 [file pharmaceuticals-19-00975-s001.zip › pharmaceuticals-4280917-supplementary.pdf]

Table S1. Complete equations for the studied independent variables

Variable	Equation	R^2	Adj R^2	MS R
Span	Y_1=0.8852+0.0175X_1+0.0005X_2-0.0270X_1X_2	0.0149	0	0.27753
Yield	Y_2=47.4040-5.2775X_1+12.7875X_2+10.3675X_1X_2	0.5769	0	876.885
Porosity	Y_3=0.5736-0.0070X_1-0.3925X_2+0.0495X_1X_2	0.78939	0.15755	0.16708
Hardness	Y_2 = 6.6016 - 1.4345X_1 + 2.3105X_2 – 1.0430X_1X_2	0.9940	0.97581	0.20645

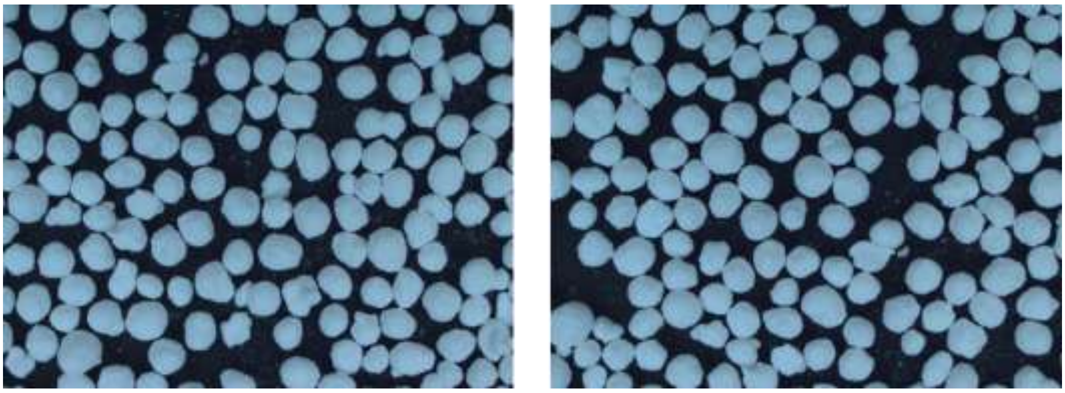

Figure S1. Microscopical images of Sample 1

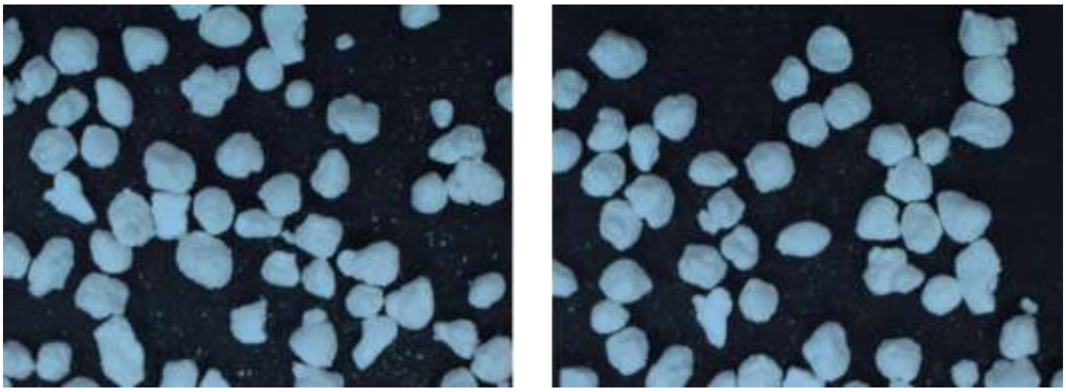

Figure S2. Microscopical images of Sample 2

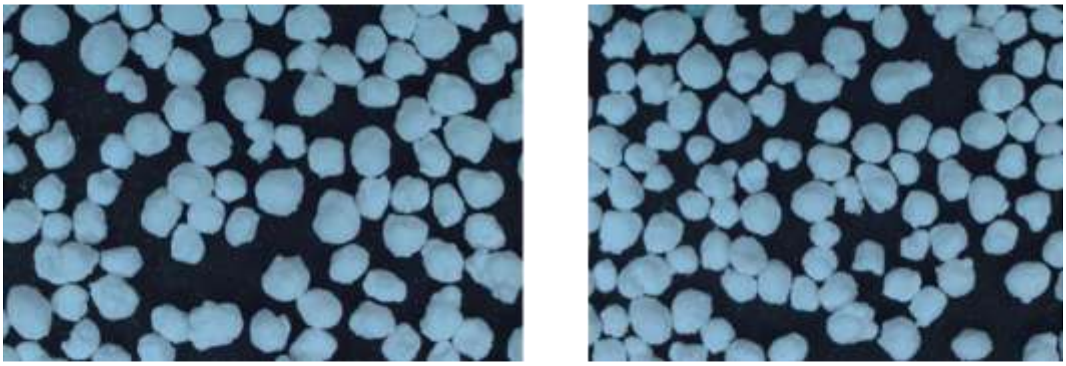

Figure S3. Microscopical images of Sample 3

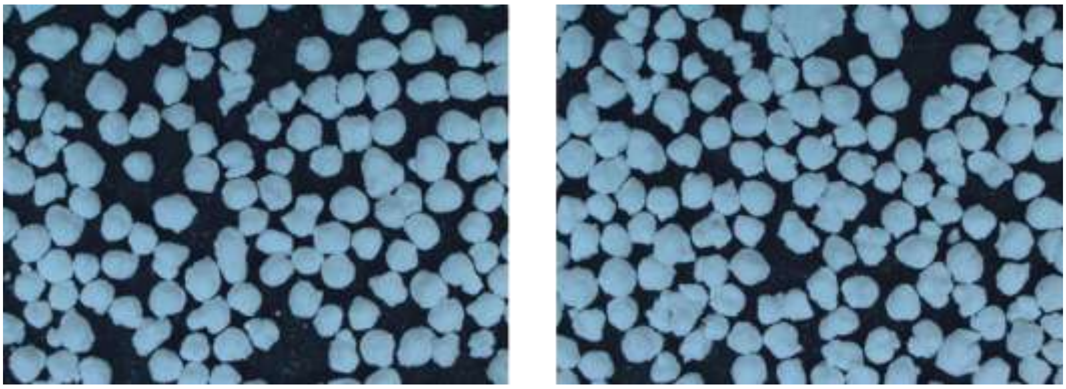

Figure S4. Microscopical images of Sample 4

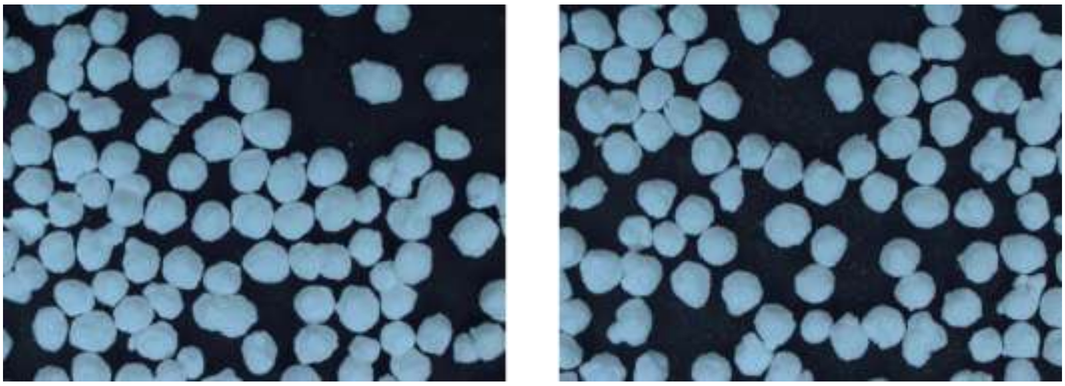

Figure S5. Microscopical images of Sample 5

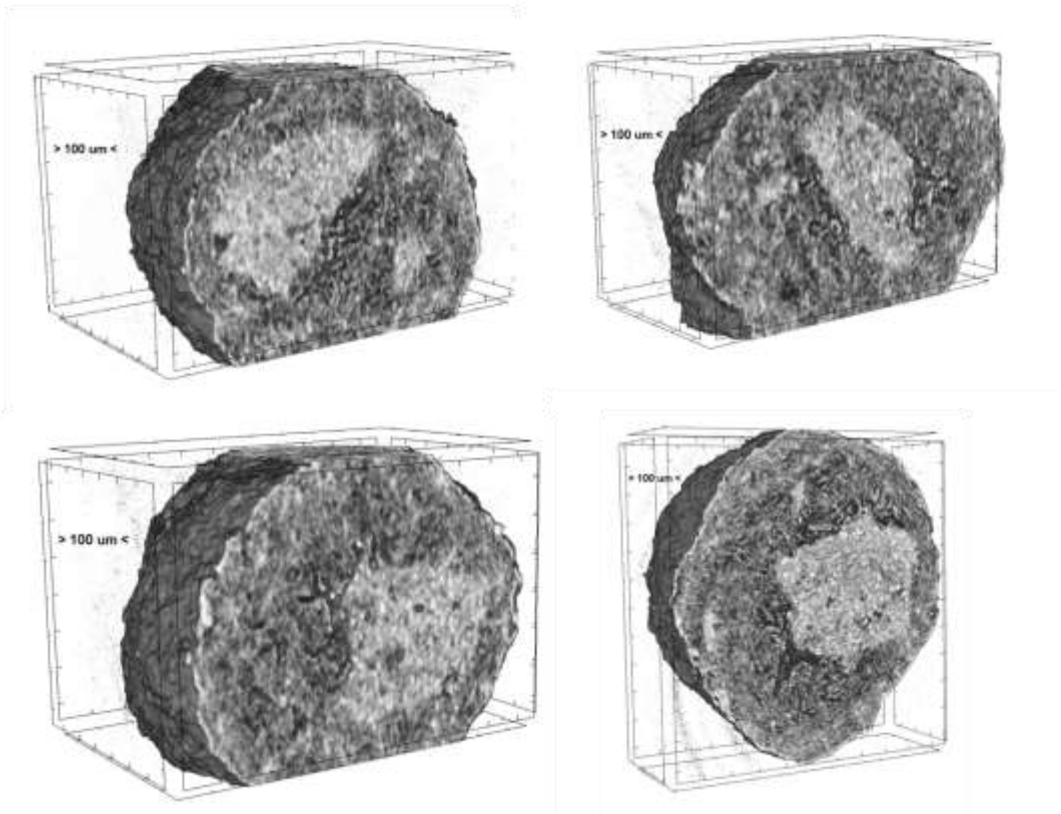

Figure S6. Micro-CT images of granules from Sample 1

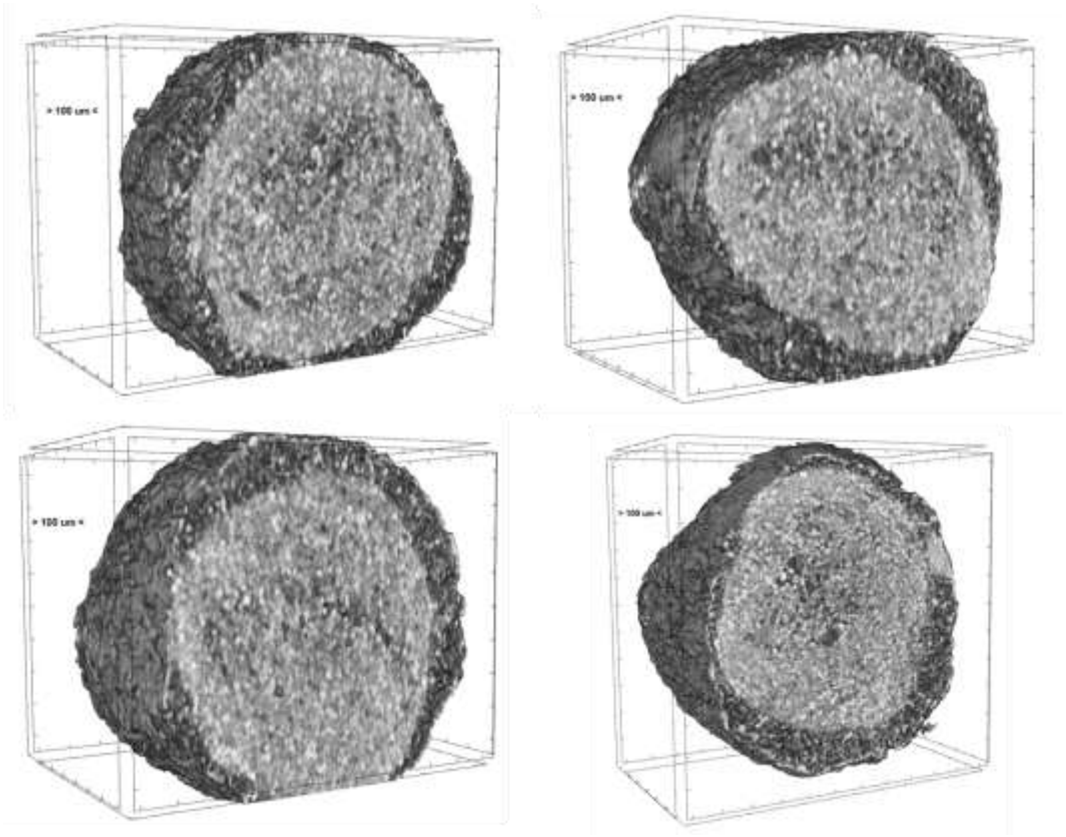

Figure S7. Micro-CT images of granules from Sample 2

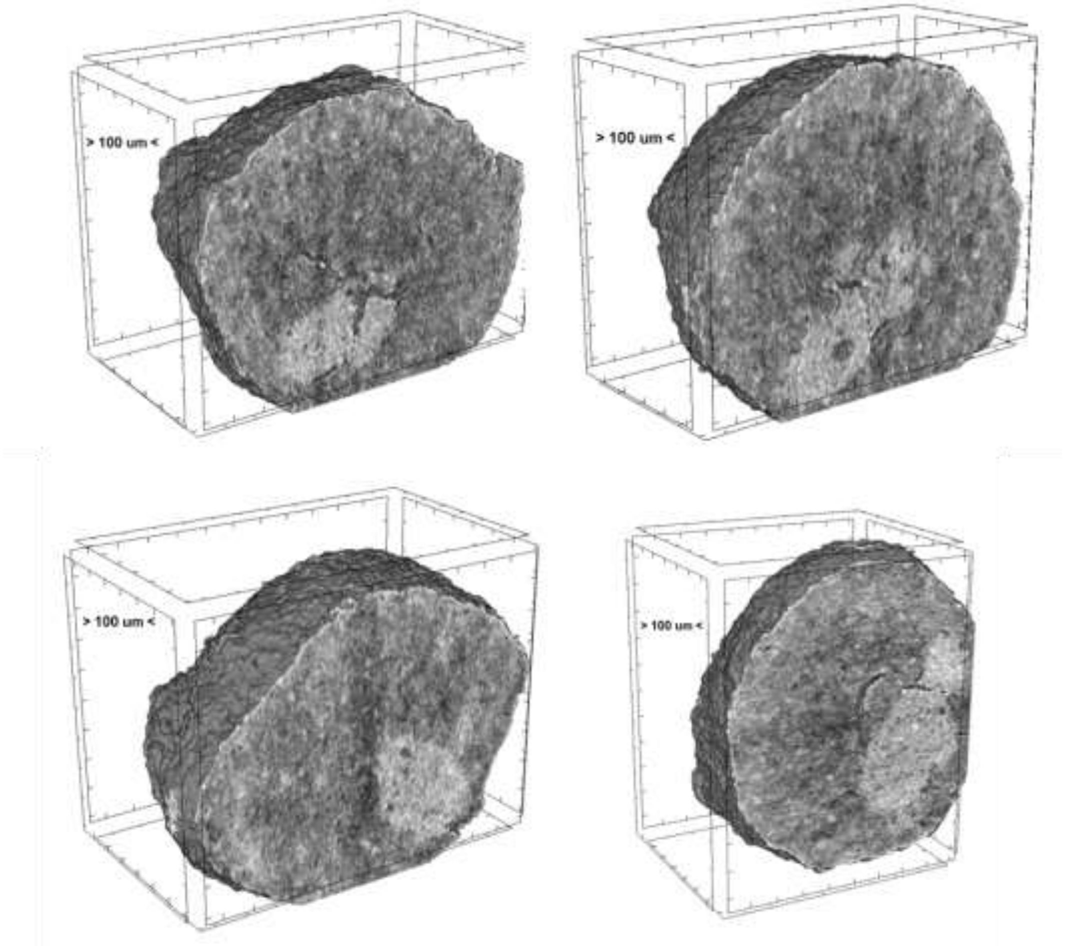

Figure S8. Micro-CT images of granules from Sample 3

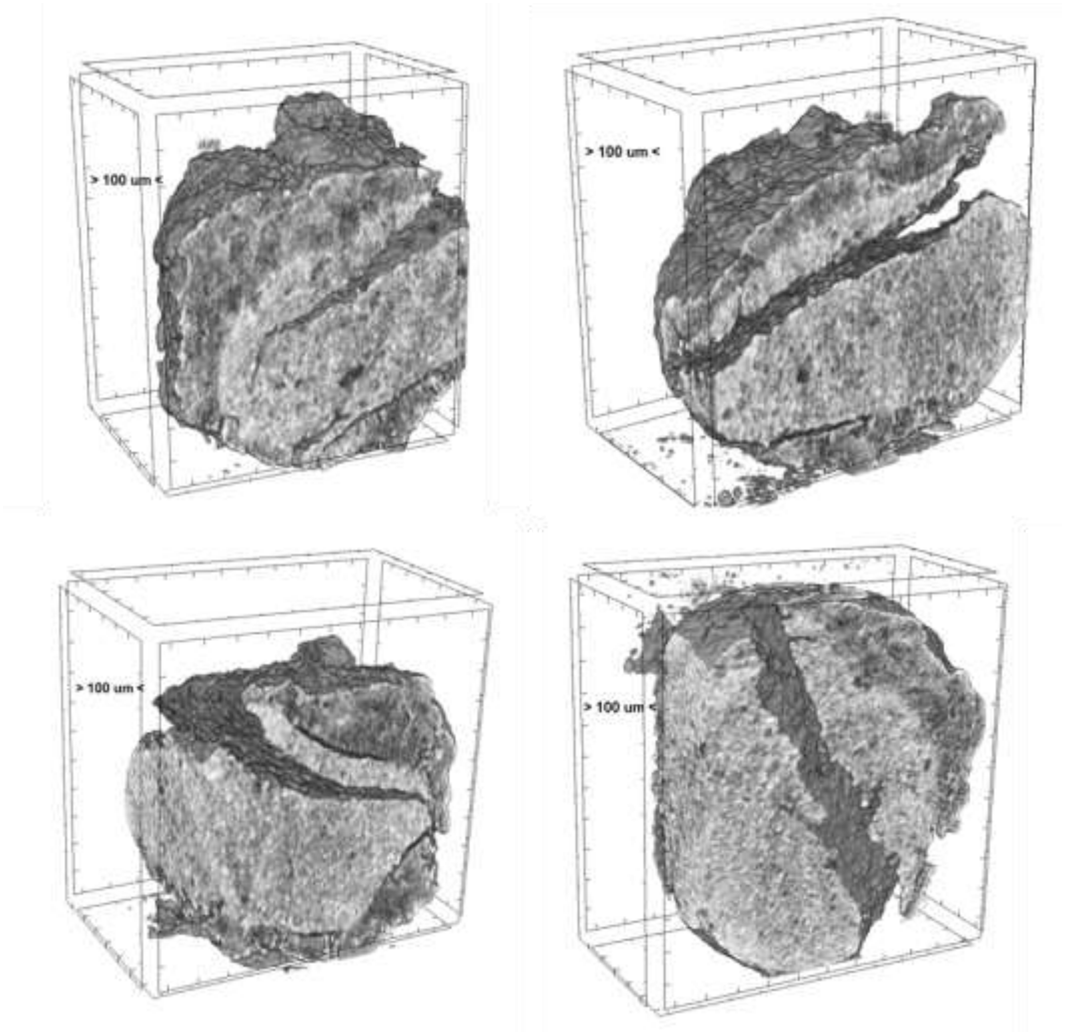

Figure S9. Micro-CT images of granules from Sample 4

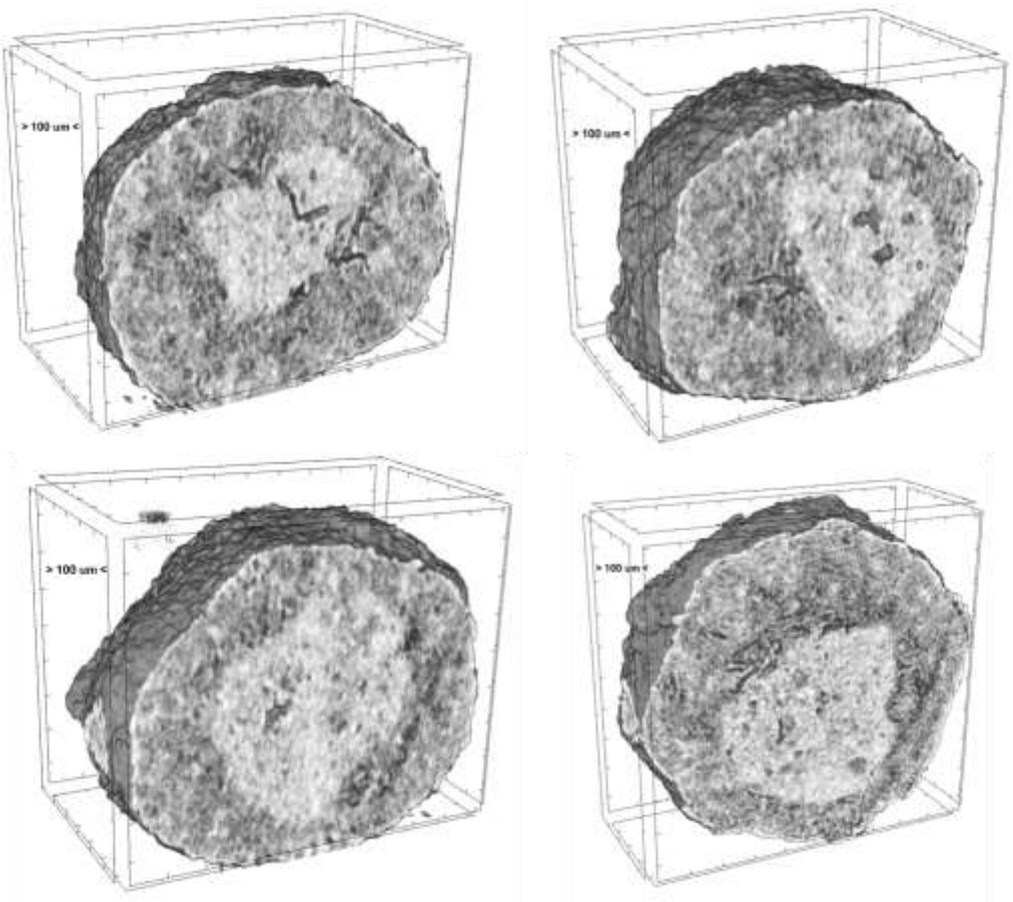

Figure S10. Micro-CT images of granules from Sample 5
